# Neurodegenerative Diseases: Molecular Mechanisms and Therapies

**DOI:** 10.3390/ijms241813721

**Published:** 2023-09-06

**Authors:** Zhi Dong Zhou, Alexandre Hiroaki Kihara

**Affiliations:** 1 National Neuroscience Institute of Singapore, 11 Jalan Tan Tock Seng, Singapore 30843, Singapore; 2 Signature Research Program in Neuroscience and Behavioral Disorders, Duke-NUS Graduate Medical School Singapore, 8 College Road, Singapore 169857, Singapore; 3 Neurogenetics Laboratory, Universidade Federal do ABC, São Bernardo do Campo 09606-045, SP, Brazil; 4 Center for Mathematics, Computing and Cognition, Universidade Federal do ABC, São Bernardo do Campo 09606-045, SP, Brazil

Neurodegenerative diseases are characterized by the progressive degeneration or death of neurons in the central or peripheral nervous system. Genetic, environmental, and lifestyle factors all contribute to neurodegenerative diseases [1,2]. Although some neurodegenerative diseases can be managed with treatments, and the progression of the disease may be slow, many remain incurable [3]. Common underlying processes contribute to the degeneration of neurons, but the molecular mechanisms of neurodegenerative diseases are complex and varied, and they can differ depending on the specific conditions [4]. Neurodegenerative processes and neuronal disorders also occur in the spinal cord [5], retina [6], and enteric nervous system [7], eventually reflecting and/or affecting what occurs in the brain. The consequences of these conditions are often a gradual decline and abnormality in perceptual, cognitive, motor, behavioral, and social abilities. Neurodegenerative diseases pose a significant public health threat. These diseases commonly occur in elderly individuals and their prevalence increases with age. The increasing lifespan and decreasing fertility rate lead to an increase in the median age prevalence of these disorders and aggravated health and economic burdens to society.

Long-lived people have a higher concentration of the longevity-associated variant (LAV) homozygous genotype in member 4 of the bactericidal/permeability-increasing fold-containing family B (BPIFB4) gene [8]. It was previously shown that LAV-BPIFB4 prevented Huntington’s disease (HD) progression in mice [9]. The production of mutant Huntingtin protein, which exhibits pathological lengthy polyglutamine repeats and toxic consequences, results in HD. In this context, there is a substantial clinical need yet unmet, as there are currently no clinically validated treatment medicines or therapies that can slow or stop neurodegeneration and disease progression in HD. In this Special Issue, Cattaneo et al. demonstrate that LAV-BPIFB4 generates neuroprotection in the striatum-derived STHdh cell line, an in vitro model of HD [10]. 

The use of mutant cell lines enables the study of the underlying pathophysiological processes causing neurodegeneration, such as oxidative stress. Besides the NADPH oxidase 2 (NOX2/gp91) gene [11,12], other NADPH oxidase family members (NOX 1, 3–5, DUOX1, and DUOX2) have been implicated in the production of reactive oxygen species (ROS) [13]. Interestingly, the NADPH oxidase organizer 1 (NOXO1) is essential for the organization and formation of the NOX1-dependent NADPH oxidase complex. Benssouina et al. demonstrate that, in a mutated NOXO1 colorectal cancer cell line with mutated D-box, there is an increase in ROS production and cytotoxicity through NOX1 activity, affecting the mitochondrial organization. These effects were observed with the translocation of the mutated protein from the soluble membrane fraction to a cytoskeletal insoluble fraction [14]. The D-box of NOXO1 protein seems to maintain the balanced distribution of Noxo1 between membrane and cytoskeleton, which is vital to Nox1-dependent NADPH oxidase activity, ROS generation, and cytotoxicity. 

Another line of research that involves the use of in vitro cell lines is related to Parkinson’s disease (PD). In a familial type of PD, loss-of-function mutations of the E3 ligase Parkin likely result in the accumulation of aberrant mitochondria and neurodegeneration, due to impaired polyubiquitination of defective mitochondria for subsequent mitophagy clearance. However, researchers’ attention has recently been directed towards Parkin’s role as a redox molecule. Using cell culture, Ardah et al. overexpressed several combinations of Parkin and its substrates FAF1, PINK1, and ubiquitin to ascertain the function of Parkin as a redox molecule in the mitochondria [15]. Unexpectedly, these authors found that the Parkin monomer was not recruited to aberrant mitochondria but instead self-aggregated into the inner and outer membranes of the mitochondria, thus becoming insoluble. Parkin overexpression alone produced Parkin protein aggregates without self-ubiquitination, which triggered autophagy to clear away damaged mitochondria. They concluded that the polyubiquitination of Parkin substrates on the mitochondria is not indispensable for the mitophagy clearance of defective mitochondria.

Indeed, PD pathophysiology has garnered attention in the scientific community, with recent reports generating original research and reviews. It is established that α-synuclein (αSyn) aggregates and Lewy body formation are some of the pathological features of PD. Regarding pathological features beyond those associated with PD, Graves et al. examined the reciprocal interaction between conformational strains of Syn aggregates and the cellular environment, discussing recent evidence for its participation in other neurodegenerative diseases [16]. The pathological aggregation of αSyn protein has also been identified in dementia with Lewy bodies (DLB), multiple system atrophy (MSA), Alzheimer’s disease (AD), brain traumatic injuries, and neurodegeneration with brain iron accumulation (NBIA). Diseases associated with αSyn protein aggregation are referred to as synucleinopathies, highlighting the potential common pathological events and disease mechanisms in these disorders.

A crucial element in the pathophysiology of PD is the self-association of amylogenic proteins to form fibril structures. Recent studies have suggested the interruption of fibrils with an external electric field as a non-invasive strategy with a few advantages over other techniques for potential clinical applications. Razzokov et al. performed molecular dynamics (MD) simulations on extremely hazardous α-synuclein fibrils to obtain a molecular-level understanding of fibril disruption mechanisms. Their findings revealed that the applied external electric field significantly alters the structure of αsyn fibrils, in addition to highlighting the critical electric field intensity at which the hydrophobic core of αsyn fibrils can be fully opened [17].

The human cystatin C (HCC) amyloidogenic protein uses a domain-swapping process to produce dimers and higher oligomers such as trimers, tetramers, and others. Wojciechowska et al. characterized the HCC oligomeric form observed in solutions with pH levels ranging from 2.2 to 10.0 and in environments that encouraged oligomerization. Size exclusion chromatography, dynamic light scattering, and small-angle X-ray scattering were used to characterize the oligomeric forms of HCC generated under various conditions. It was found that HCC has a strong capacity to form dimers at pH 4.0–5.0 and tetramers at lower pH (2.3 or 3.0). When pH is higher than 6.0, HCC can remain in monomeric form. However, as the environment changes from acidic to neutral, HCC tetramers break down into dimers. They concluded that the interaction of dimers generates HCC tetrameric forms without a domain-swapping mechanism, as evidenced by the breakdown of tetramers into dimers at pH 7.4 [18].

Another research topic of high interest for studies on PD and AD is related to exosomes. Exosomes play a specific function in the pathophysiology, diagnosis, and prognosis of AD and PD, but their pathophysiological role is not yet fully understood. In a recent review, He et al. discussed the biosynthesis, components, uptake, and functions of exosomes, as well as their pathological roles in AD and PD [19]. In addition to highlighting the use of exosomes as drug carriers for therapies in the central nervous system, the authors provided an overview of recent advances in the clinical implications of exosomes in the treatment and diagnosis of AD and PD.

One of the major challenges in the treatment of neurodegenerative diseases involves finding measures that can facilitate drug penetration through the blood–brain barrier (BBB). The main in vitro BBB models developed thus far for investigating the BBB barrier qualities of the cerebral vasculature are summarized and discussed by Chaulagain et al. They describe multiple existing in vitro models, such as 3D organoid models, microfluidic models, 2D transwell models covering single-layer and co-culture models, and 2D transwell models, together with their construction, permeability measurement, uses, and limitations [20]. Recent achievements and future challenges are discussed and highlighted. 

Fasudil, a pan Rho-associated coiled-coil protein kinase (ROCK) inhibitor that dilates blood vessels, has been used for postcerebral stroke. More recently, the use of this medication has been suggested for the treatment of neurodegenerative diseases. Killick et al. conducted a global gene expression analysis in the brains of mice using an AD mouse model, in which fasudil was administered via peripheral intraperitoneal injection [21]. The authors compared the fasudil-induced distinct transcriptional profile in the AD mice model with profiles derived from meta-analyses of other neurodegenerative disorders. The results of their analyses reveal that fasudil stimulates gene expression, in contrast to the gene expression profiles in postmortem brains of patients with neurodegenerative diseases. According to the results of their pathway enrichment analysis, fasudil strongly drives pathways in a reverse manner to disrupted pathways in AD and PD. These findings support the potential repurposed use of fasudil for AD and PD patients, which warrants future investigations.

Numerous lysosomal storage disorders and human neurodegenerative diseases have autophagic dysfunction. This deficiency of lysosome function could exacerbate metabolite buildup and lysosomal distress, subserving the emergence of a neurodegenerative phenotype. Krabbe disease (KD) has recently been linked to abnormalities in the lysosome and autophagy pathways. Large-scale demyelination and dysmyelination are hallmarks of KD, which is triggered by a genetic defect in the lysosomal enzyme galactocerebrosidase (GALC) gene. Galactosylceramide, psychosine, and secondary substrates such as lactosylceramide accumulate due to the loss of function of GALC. Papini et al. used an in vitro cellular starvation model to induce autophagy and observed the cellular reactions and molecular events in patient-isolated fibroblasts [22]. They revealed that the inhibitory AKT-induced beclin-1 phosphorylation and the BCL2–beclin-1 complex jointly contribute to repressing the generation of autophagosomes under starvation [22]. The KD fibroblast model showed autophagic response defects, which could be linked to upregulated AKT activity [22]. These occurrences were not relevant to psychosine accumulation, which was suggested as a key event leading to autophagic dysfunction in KD. 

This Special Issue highlights researchers’ continuous investigations of the underlying pathophysiological mechanisms and potential treatments of neurodegenerative diseases. Besides the comprehensive understanding of molecular mechanisms subserving the onset and progression of neurodegenerative diseases, early diagnosis and lifestyle factors such as a healthy diet, exercise, and mental stimulation will also contribute to symptom management and improvement in the life quality of the affected individuals.
